# The ADHD teen integrative data analysis longitudinal (TIDAL) dataset: background, methodology, and aims

**DOI:** 10.1186/s12888-020-02734-6

**Published:** 2020-07-08

**Authors:** Margaret H. Sibley, Stefany J. Coxe

**Affiliations:** 1 Department of Psychiatry and Behavioral Sciences, University of Washington School of Medicine, Seattle Children’s Research Insitute, 2001 8th Ave., Suite 400, Seattle, WA 98117 USA; 2 Department of Psychiatry & Behavioral Health, Florida International University, 11200 SW 8th Street, Miami, FL 33199 USA; 3 Department of Psychology, Florida International University, 11200 SW 8th Street, Miami, FL 33199 USA

**Keywords:** ADHD, Longitudinal data, Adolescence, Intervention

## Abstract

**Background:**

The Attention Deficit Hyperactivity Disorder (ADHD) Teen Integrative Data Analysis Longitudinal (TIDAL) dataset integrates data from four randomized trials.

**Method:**

Participants with ADHD (*N =* 854; 72.5% male, 92.5% racial/ethnic minority, ages 10–17) were assessed three times across 12 months. Data includes parent, self, and teacher ratings, observations, and school records. The battery was harmonized using an Integrative Data Analysis (IDA) approach to form variables that assign unique values to all participants.

**Results:**

The data will be used to investigate: (1) profiles that organize the heterogeneous population into clinically meaningful subgroups, (2) whether these profiles predict treatment response, (3) heterogeneity in treatment response and variables that predict this response, (4) how treatment characteristics and adjunctive supports predict treatment response, and (5) mediators of treatment and whether these mechanisms are moderated by treatment characteristics.

**Conclusions:**

The ADHD TIDAL Dataset will be openly shared with the field to maximize its utility.

## Background

The teenage years are a critical period for ADHD intervention. Longitudinal studies indicate a close relationship between adolescent functioning and ADHD persistence into adulthood [1–3]. Moreover, high neuroplasticity during adolescence may potentiate skill learning that maintains long-term [4], while negative adolescent life events (i.e., legal troubles, dropout, teen pregnancy) derail adult trajectories [1]. The last 10 years witnessed a proliferation of empirically-supported psychosocial interventions for adolescents with ADHD [5, 6]. These interventions teach adolescents compensatory skills to mitigate the effects of executive functioning deficits on daily life while training adult stakeholders to use age-appropriate contingency management to reduce the effects of rewards processing deficits [7, 8]. Psychosocial treatments are a strong developmental-fit to the adolescent period because: (1) adolescents with ADHD often dislike stimulant medication and desist use [9, 10] and (2) adolescent psychosocial treatments outperform medication in mitigating ADHD-related impairment, showing medium to large effects [6].

Despite their efficacy, implementation and utilization of adolescent ADHD treatments are poor [11–14]. Thus, efforts are needed to identify patient and service delivery barriers and facilitators. Understanding who fails to engage in treatment and why will signal opportunities to decrease treatment disparities.

The clinical profiles of adolescents with ADHD are highly heterogeneous [15–17]; yet, almost nothing is known about which treatments work best for whom, when, where, and how. Practitioners have few guidelines for treatment optimization—critical choices that influence a treatment credibility and patient retention in care [18]. Studies investigating questions of moderation and mediation are necessary to inform nuanced clinical care decisions [19, 20]. Unlike treatments for childhood ADHD [21], there are almost no large-scale trials of treatment for adolescent ADHD. Much could be gleaned from a large-scale investigation of mechanisms of change in adolescent ADHD treatment, identification of treatment-relevant phenotypes that influence response, and tracing the role of adjunctive supports (i.e., medication, parent involvement style, school accommodations) in enhancing or detracting from psychosocial treatment. Rich clinical information could be derived from investigating how treatment response varies as a function of person- and service delivery-level variables [22].

Pursuing these aims require person-level approaches (i.e., mixture modeling, latent class analysis) [23]. As the field of child and adolescent mental health moves toward precision medicine [24], personal-level analyses are essential to ensuring maximally effective care. Precision medicine studies have led to important advances in the treatment of childhood conduct problems [25] and adult depression [19], among other disorders. These analyses require large sample sizes that have not yet been available in the treatment of adolescent ADHD.

### Overview of the ADHD TIDAL dataset

To increase data resources in the field of adolescent ADHD, we constructed the ADHD Teen Integrative Data Analysis Longitudinal (ADHD TIDAL) dataset. The ADHD TIDAL dataset will be openly shared with the field. We integrated data from four randomized trials (*N =* 854; ages 10–17) [26–29] that cumulatively tested the comparative efficacy of unique five treatment conditions (i.e., evidence-based parent-teen therapy, group parent training and teen organization skills training, intensive summer treatment, usual care psychotherapy, no treatment) and included data from three unique settings (university clinic, schools, community mental health). These data were combined using an Integrative Data Analysis [30] framework, resulting in a comprehensive dataset. In the current paper, we describe the dataset, our methodology, planned analyses to pursue a linked series of person-level research questions, and additional research questions that scientific investigators might pursue using the dataset.

### Construction and content

#### Integrative data analysis

Integrative data analysis is a relatively new technique that allows researchers to pool raw data from multiple studies to produce cumulative scientific knowledge [30]. IDA differs from more well-known techniques for combining information, such as meta-analysis, in that IDA analyzes pooled raw data from each study rather than summary statistics. IDA has several advantages over separate analysis of each study [31], including increased statistical power, management of sample heterogeneity, and increased frequency of low base-rate behaviors. IDA framework required us to: (1) code study characteristics, (2) harmonize measures and/or create commensurate measures, and (3) select a type of IDA. The four included studies vary on a variety of characteristics (i.e., treatment and comparison groups, referral source, and time of year for treatment). A major task for IDA is carefully coding each study on these characteristics. Per Hussong and colleagues [31], we coded each study based on sampling approach, history, design characteristics, and measurement. Codes are integrated into analyses as dictated by research questions.

To conduct an analysis on the combined dataset, the same variables must be present in some form in all studies. The four studies were conducted by the same investigators, so many measures of interest are common across studies (e.g., Diagnostic Interview Schedule for Children) [32] and require no additional work to use in an IDA (though IDA provides an opportunity to explore measurement invariance across studies). Other measures, such as parent depression (e.g., Patient Health Questionnaire-9,Symptom Checklist-90-Revised, World Health Organization Quality of Life) [33–35] and ADHD symptoms (i.e., DSM-IV-TR vs. DSM-5 symptom checklists) [6] are not identical, requiring development of commensurate measures. Commensurate measures typically involve item response theory (IRT) analysis to create common scale scores [36, 37].

IDA allows for either random or fixed effects models, depending on the number of studies and whether a study is conceptualized as a random sample from the population of interest. Fixed effects IDA conceives of each study as a known, specific sample from the population and can be conducted with as few as two studies. Random effects IDA conceives of each study as a random sample from the population and requires a minimum of 20 to 30 studies. The ADHD TIDAL dataset includes four studies, so all analyses are conducted within a fixed-effects IDA framework. This means that dummy codes indicating study membership are included in each analysis to account for differences between studies. We interpret all results within a fixed effects IDA framework indicating that: (1) we can only make inferences back to the specific studies, not to similar studies on this population and (2) we cannot fully disaggregate some between- and within-study effects, due to study-specific code variables in analyses (i.e., whether variance attributed to “summer treatment program” is due to time of year or dose).

### Study designs

#### Common elements

Our four studies were chosen for the IDA because they shared common characteristics that promoted successful harmonization. From 2010 to 2019, the research team conducted seven longitudinal treatment outcome studies of psychosocial treatment for adolescents with ADHD. We sought to include studies in the IDA that: (1) included participants from the large local school district with standardized attendance, grades, and disciplinary data, (2) possessed inclusion criteria that all participants meet DSM criteria for ADHD during a comprehensive psychiatric evaluation that included a structured parent interview (Diagnostic Interview Schedule for Children; DISC) [32] and parent and teacher symptom and impairment ratings that were integrated and reviewed by licensed clinical psychologists; (3) possessed a randomized controlled trial design; and (4) included baseline, post-treatment, and follow-up data points. Based on these criteria, two of the research teams studies were excluded from the IDA because they did not possess a follow-up data point and one was excluded because it did not possess a randomized control group. Comparison of the four included studies indicated additional common features that suit the IDA framework: (1) Autism Spectrum Disorders were exclusionary in all studies (participants with other comorbidities were included) and (2) all study participants were permitted to continue stimulant medication and special education interventions at school. These adjunctive treatments were monitored carefully and can be included as time varying covariates in analyses. An overview of study design features is provided in Table 1.

**Table 1:** Study Characteristics of the ADHD TIDAL Dataset

	Study A	Study B	Study C	Study D
Sample Size	*N* = 128	*N* = 325	*N* = 123	*N* = 278
Referral source	Parent/Teacher Clinic Referral	School-Referred	Parent/Teacher Clinic Referral	Community Mental Health
Setting	University Clinic	School	University Clinic	Community Mental Health
Time of Year	Fall or Spring	Summer	Fall, Winter, or Spring	Fall, Winter, or Spring
Treatment Duration	10 weeks	8 weeks	8–10 weeks	10 weeks
Treatment Arms	STAND (*n* = 67) NOTX (*n* = 61)	STP-A (*n* = 109) STAND-G (*n* = 109) NOTX *(n* = 107)	STAND (*n* = 63) STAND-G (*n* = 60)	STAND (*n* = 138) UC (*n* = 140)
Clinician Type	Graduate and Post-Graduate Trainees, Masters Level	School District Staff, College Student Interns	Graduate Trainees, Licensed Clinicians	Community Mental Health Practitioners
Start of study	Fall 2011	Spring 2012	Spring 2014	Fall 2015
BL to POST	6 months	6 months	6 months	4 months
BL to FU	12 months	12 months	12 months	10 months

*Note. STAND* Supporting Teens’ Autonomy Daily; *STAND-G* STAND-Group; *STP-A* Summer Treatment Program-Adolescent; *NOTX* No treatment provided by research team; *UC* Usual Care in Community Mental Health. *BL* Baseline, *POST* Post-treatment; *FU* Follow-up

#### Study A

In study A (see Table 1) [26] middle school students were randomized to Supporting Teens’ Autonomy Daily (STAND) [38] in the university clinic or a treatment as usual control group in which no treatment was offered to participants by the research team. Admission to the study used a cohort design with students receiving 10 weeks of treatment in the spring (cohort 1 and cohort 2) or the fall (cohort 3) of the academic year. At post-treatment, all participants had data from at least one source and 95% of participants had data from at least two sources. At follow-up, 97% had data from at least one source and 87% had data from at least two sources [26].

#### Study B

Study B (see Table 1) [27] randomly assigned rising 6th or 9th graders with ADHD to the intensive Summer Treatment Program-Adolescent (STPA) or group STAND (STAND-G). School district personnel delivered the STP-A, which was held in district schools with bus transportation provided. In the fourth year of the study, a no treatment comparison group of 107 students was recruited and tracked using the same assessment schedule to contextualize group differences between the two active treatment arms. Retention converged at 90–95% across sources, time points, and groups [27].

#### Study C

Middle or high school students with ADHD (see Table 1; *N* = 123) were randomly assigned to STAND or STAND-G using a stratified randomization procedure within study wave [28]. Study enrollment occurred in six waves with approximately ten participants per modality per wave. Recruitment occurred across 24 months with each wave occurring approximately 4 months apart. Treatment was delivered on a rolling basis throughout the academic year, with a pause in recruitment and treatment over the summer months. Retention was strong at post-treatment (95.1–97.6%) and follow-up (85.4–91.9%).

#### Study D

This trial (see Table 1) tested the effectiveness of STAND versus Usual Care in a sample of middle and high school students with ADHD (*N =* 278) who were incoming patients at four community mental health clinics. Over 3 years, treatment was provided by agency employees who were randomly assigned to receive STAND training and supervision or treat cases using UC practices. Adolescents were also randomized to STAND vs. UC. Retention was 99.3% at post-treatment and 97.5% at follow-up (data from at least one informant).

#### Heterogeneity

Combining the data across the four studies increases sample heterogeneity and allows for examination of between-study heterogeneity. Heterogeneity (i.e., variance) is an advantage when trying to find relationships between variables. Particularly in studies of clinical populations, restricted range can reduce statistical power and impede detection of relationships between variables. The larger, more heterogeneous combined sample improves statistical power. In addition, traditionally under-represented groups (e.g., girls with ADHD) and behaviors (e.g., conduct disorder) are well-represented in the IDA sample.

While the four studies are similar in scope, they also differ in several ways. Study B includes school-referred youth, study D includes patients in community agencies, and studies A and C included patients at a university clinic. Studies A and B utilize a no treatment control group; studies B, C, and D compare active treatments, including therapist-selected intervention in community mental health (i.e., agency usual care). Study B included summer treatment, while studies A, C, and D included treatment delivered at various points during the school year.

#### Demographic Characteristics

Demographic characteristics of the full sample (*N* = 854) are presented in Tables 2 and 3. The larger combined dataset allows for examination of typically low base-rate sample characteristics. There are many clinically-meaningful behaviors that are infrequently exhibited, even in clinical samples of adolescents with ADHD, such as superior IQ, predominantly hyperactive/impulsive presentation, or conduct disorder. Tables 2 and 3 illustrate that typically underrepresented patient populations, such as females and African-Americans with ADHD can be pooled across studies to create cell sizes that are now sufficient for analysis.

**Table 2:** Demographic Characteristics of the ADHD TIDAL Dataset (N = 854)

Adolescent Demographic	
Age *M* (*SD*)	13.33 (1.58)
Gender % (n)	
Male	72.5 (619)
Female	27.5 (235)
Race/Ethnicity % (n)	
White Non-Hispanic	7.5 (64)
African-American	13.5 (115)
Hispanic Any Race	77.2 (659)
Other	1.8 (16)
Number of Siblings *M* (*SD*)	1.80 (1.39)
Parent Demographic	
Primary Caregiver % (n)	
Mother	89.2 (762)
Father	8.2 (70)
Other	2.6 (22)
Parent Education Level % (n)	
High School or less	21.2 (181)
Some college or Associate’s	29.9 (255)
Bachelor’s degree	31.1 (266)
Master’s degree or higher	17.4 (149)
Undisclosed	0.7 (6)
Single Parent % (n)	39.2 (334)
Parent Primary Language % (n)	
English	74.4 (635)
Spanish	25.6 (219)

**Table 3:** Clinical Characteristics of the ADHD TIDAL Dataset

Estimated Full Scale IQ *M* (*SD*)	96.59 (13.13)
ADHD Diagnosis % (n)	
ADHD-Predominantly Inattentive Type	46.6 (398)
ADHD-Combined Type	53.0 (453)
ADHD-Predominantly H/I	0.4 (3)
Oppositional Defiant Disorder % (n)	42.5 (363)
Conduct Disorder % (n)	5.7 (49)
Baseline ADHD Medication % (n)	35.9 (307)
School Accommodations % (n)	
Individualized Education Plan	33.4 (285)
Section 504 Plan	18.5 (158)
None	45.9 (392)
Unreported	2.2 (19)
Class Placement % (n)	
Gifted	17.0 (145)
Regular	68.5 (585)
Regular + Special Education/Inclusion	12.1 (103)
Self-Contained Special Education	1.8 (15)
Unreported	0.7 (6)

### Treatment conditions

#### Supporting teens’ autonomy daily (STAND)

STAND is an engagement-focused psychosocial treatment for adolescent ADHD. STAND is manualized and consists of 10 weekly 60-min sessions attended by the adolescent and parent. Skill instruction is blended with Motivational Interviewing [39] and guided parent-teen behavioral contracting [40]. Treatment targets family, behavioral, and academic impairment. In the engagement phase, MI is used to increase awareness of personal values and goals, identify strengths, and recognize ways to achieve goals by acting consistently with values. The skills phase is designed to teach parent-teen communication, parent behavioral strategies, and organization, time management and planning skills applied to homework, school, and chores. Planning sessions teach families to integrate skills into a daily routine, transfer new habits to school settings, and build a final parent-teen contract. In all studies, STAND was offered in either English or Spanish. Therapists are offered 3 days of training.

#### STAND-group

STAND-Group (STAND-G) is manualized and consists of eight 90-min weekly sessions attended by the adolescent and parent [38]. Parents and adolescents meet in separate groups for the first 75 min and a blended parent-teen group for the final 15 min. Parent training employs the community-based model [41], alternating between didactic instruction, and small and full group discussions. Parents are exposed to the same skills as in STAND, including how to monitor academics, set a daily routine, apply behavioral principles to homework, and create a parent-teen contract. The adolescent skills group alternated didactic instruction (e.g., introduction of a new skill), hands on activities (e.g., organizing one’s backpack with a peer), and discussion exercises (e.g., debating the pros and cons of writing in a planner). Adolescents and parents are given suggested exercises to practice skills at home between sessions (e.g., negotiating a homework plan, organizing one’s backpack and scheduling parent backpack checks). Therapists receive 1 day of training prior to implementing treatment. In study B, school consultation was offered in addition to STAND-G; however, almost no participants received a meaningful dose of this intervention component [14].

#### Summer treatment program-adolescent

The 8-week STP-A [27] includes 45 h of youth directed treatment per week. Intervention includes rotating group modules targeting materials management, time management, planning, homework completion, note-taking, study skills, writing skills, self-monitoring, decision-making (including LifeSkills© Training) [42], social pragmatics, and independently managing responsibilities in a vocational program. Contingency management is incorporated to enhance adolescent motivation to practice skills. A two-week training includes didactics, discussions, tests, role-playing, and practice. Each day, lead counselors telephone parents to provide a verbal summary of the adolescent’s performance on daily treatment goals and offer coaching on home contingency management. Parents also receive an eight-week manualized parent training curriculum [43] as described for STAND-G.

#### Usual care

Usual Care (UC) therapists at community mental health agencies were instructed to treat study cases using usual procedures in the agency and the treatments they believed would be most effective. They received weekly supervision from agency supervisors according to typical agency practices. Complex analyses of UC practices have been proposed as a future direction; at present, UC psychotherapy for adolescent ADHD remains a black box.

#### No treatment

In Study A, a treatment as usual comparison group was offered no treatment by the study team. In Study B, an untreated comparison group was followed in the fourth year of the study. In these conditions, participants were permitted to pursue naturalistic treatment in their communities.

#### Measures and available data

Table 4 lists available data for each of the measures by administration schedule.

**Table 4:** Measures Administration and Available Data in ADHD TIDAL Dataset

	Baseline					Post-Treatment					Follow-up				
	A	B	C	D	*N*	A	B	C	D	*N*	A	B	C	D	*N*
Adolescent Academic Problems Checklist															
Self	X	X	–	–	453	X	X	–	–	416	X	X	–	–	411
Parent	X	X	X	X	852	X	X	X	X	776	X	X	X	X	735
Teacher	X	X	X	X	844	X	X	X	X	798	X	X	X	X	759
Conflict Behavior Questionnaire															
Self	X	X	X	X	850	X	X	X	X	780	X	X	X	X	757
Parent	X	X	X	X	853	X	X	X	X	780	X	X	X	X	733
Disruptive Behavior Disorder-Rating Scale															
Parent	X	X	–	–	452	X	X	–	–	412	X	X	X	–	389
Teacher	X	X	–	–	448	X	X	–	–	418	X	X	X	–	404
DSM-5 ADHD Rating Scale															
Parent	–	X^a^	X	X	582	–	X^a^	X	X	503	–	–	X	X	344
Teacher	–	–	X	X	399	–	–	X	X	381	–	–	X	X	356
Self	–	X^a^	–	X	292	–	X^a^	–	–	104	–	X^a^	–	–	104
Child Behavior Checklist	X	X	–	X		–	X	–	X		–	X	–	X	
Parent Items					731					540					518
Parent T-Scores					731					540					529
Youth Self Report	X	X		X		–			X				–	X	
Youth Items					730					544					549
Youth T-Scores			–		730		X	–		547	–	X			556
Direct Observation of Organization															
Organization Checklist	X	X	–	X	702	X	X	–	X	652	X	X	–	X	587
Daily Planner Use	X	X	–	X	651	X	X	–	X	629	X	X	–	X	594
School Records															
GPA	X	X	X	X	829	X	X	X	X	811	X	X	X	X	768
Percentage of Work Turned In	X	X	X	X	795	X	X	X	X	782	X	X	X	X	736
Average Assignment Grade	X	X	X	X	796	X	X	X	X	781	X	X	X	X	736
Average Test Grade	X	X	X	X	774	X	X	X	X	755	X	X	X	X	736
Disciplinary Incidents	–	X	–	X	599	–	X	–	X	578	–	X	–	X	567
Standardized Testing															
IQ	X	X	X	X	854	–	–	–	–	–	–	–	–	–	–
Reading Achievement	X	X	–	X	727	–	–	–	–	–	–	–	–	–	–
Math Achievement	X	X	–	X	731	–	–	–	–	–	–	–	–	–	–
Impairment Rating Scale															
Parent Academic Item	X	X	X	–	573	X	X	X	–	521	X	X	X	–	487
Teacher Academic Item	X	X	X	–	545	X	X	X	–	524	X	X	X	–	497
Parent All Items	X	X	–	–	451	X	X	–	–	407	X	X	–	–	383
Teacher All Items	X	X	–	–	432	X	X	–	–	415	X	X	–	–	391
Diagnostic Interview Schedule for Children (DISC)	X	X	X	X	854	–	–	–	–	–	–	–	–	–	–
Parent Academic Management Scale															
Items	X	X	X	X	850	X	X	X	X	777	X	X	X	X	728
Parental Involvement: Number of Hours	–	X^a^	X	X	480	–	X^a^	X	X	444	–	X^a^	X	X	435
Caregiver Strain Questionnaire	X	X	–	X	731	X	X	–	X	658	X	X	–	X	625
Parent ADHD Self-Report Scale	X	X	X	–	567	–	X^a^	X	–	218	–	–	X	–	100
Parental Psychopathology															
Symptoms Checklist-90 Revised	X	X^a^	–	–		–	–	–	–	–	–	–	–	–	–
Items					234										
T-Scores					234										
Patient Health Questionnaire-9	–	–	X	–	122	–	–	X	–	115	–	–	X	–	100
World Health Organization Quality of Life	–	–	–	X	266	–	–	–	X	251	–	–	–	X	238

^a^Indicates that scale was administered to selected cohorts and is not available for all participants in the study

#### Adolescent academic problems checklist

The self, parent, and teacher-report versions of the 24-item *Adolescent Academic Problems Checklist* (AAPC) measure observable secondary-school specific organization, time management, and planning (OTP) problems and are validated for use in samples of adolescents with ADHD [44]. The AAPC possesses two distinct factors (academic skills and disruptive behavior) and a total score, with strong internal reliability and concurrent validity [44]. One item was removed during scale development (locker organization) but is included.

#### Conflict behavior questionnaire

The parent and teen Conflict Behavior Questionnaire-20 (CBQ-20) assessed the quality of the parent-teen relationship. Respondents were asked to rate statements on a five-point scale from 1 (*strongly agree*) to 5 (*strongly disagree*) [45]. The CBQ-20 is a 20-item scale adapted from the 73-item CBQ. The CBQ-20 contains the CBQ items that best discriminated distressed and nondistressed families. It yields a single score that correlates .96 with the CBQ [45].

#### Disruptive behavior disorders rating scale

In the DSM-IV-TR era, the parent and teacher Disruptive Behavior Disorders Ratings Scale (DBD-RS) [46] measured Inattention (IN), Hyperactivity/Impulsivity (HI), ODD, and CD severity. Respondents were asked to rate symptoms as 0 (*not at all present*), 1 (*just a little*), 2 (*pretty much*), or 3 (v*ery much*). To calculate an index of symptom severity the average level (0–3) of each item on the ADHD subscales is obtained. The psychometric properties of the DBD rating scale are very good, with support for internally consistent subscales [47].

#### DSM-5 ADHD rating scale

In the DSM-5 era, IN and HI were measured using a DSM-5 ADHD Rating Scale completed by adolescents, parents, and teachers [48]. Respondents rated symptoms of ADHD as 0 (*not at all*) to 3 (v*ery much*). Symptom severity is the mean level (0–3) of ADHD subscale items. Psychometric properties of the measure are very good, with empirical support for internally consistent IN and HI subscales [48]. The DSM-5 ADHD rating scale includes the adolescent/adult symptom modifiers that were introduced in the DSM-5 [49].

#### Child behavior checklist and youth self report

The parent-reported Child Behavior Checklist (CBCL) and Youth Self Report (YSR) were administered as broadband youth psychopathology scales [49]. These scales are well-validated measures of psychosocial adjustment problems. T-scores for the full range of clinical, diagnostic, and competence scores are included in the dataset.

#### Observed organization skills

Observations of planner use assessed the degree to which students recorded homework assignments. Percentage of classes in which homework was recorded (or some indication of no homework) was calculated for the last 5 days of school. Planner use was calculated as the mean of daily scores. Photocopies or screenshots were obtained to document use. If the adolescent did not record any homework, he/she received a score of zero. This measure demonstrates high inter-rater reliability (intraclass correlation was .98 in past trials) [26]. Observations of bookbag organization were obtained using an adaptation of the Organization Checklist [50]. Trained research assistants assessed dichotomously scored items on the organization checklist such as “Is the adolescent’s bookbag free from loose papers?” Organization checklist scores are shown to correlate with teacher ratings of impairment in adolescents with ADHD [50].

#### Grades

Report cards were obtained directly from the school district at the end of each academic quarter. GPA for each quarter was calculated by converting academic grades (e.g., English, Math, Science, Social Studies) to a 5-point scale (i.e., 4.0 = A to 0.0 = F). Grades were not weighted for the difficulty of the class. GPA provides an objective and ecologically valid measure of school performance that is meaningful to parents and schools. The average grade on each completed assignment was also calculated. Assignments included any mandatory academic work turned in by the student except for tests, quizzes, and exams (i.e., homework, classwork, projects, presentations). Extra credit assignments and class participation were not counted towards this average. Missing assignments were also not weighted in the average. The average grade on each test, quiz, or exam was calculated. The percentage of assignments turned in calculated by dividing turned-in assignment count by the total number of assignments due.

#### Disciplinary incidents

The school district provided records of student disciplinary incidents at the end of each year. Counts of each type of disciplinary incident (e.g., detention, in-school suspension) were calculated and coded according to Robb and colleagues [51]. Minor disciplinary incidents included detentions, warnings, and being sent to an administrator or counselor due to behavioral issues. Major incidents included suspensions and expulsions.

#### IQ and academic achievement

The Wechsler Abbreviated Scale of Intelligence (WASI or WASI-II) was administered to participants as an index of IQ. Full-scale IQ was measured using a composite score from the Matrix Reasoning and Vocabulary subtests (Full-2) or all four subscales (Full-4) of the Wechsler Abbreviated Scale of Intelligence-2nd Edition (WASI-II) [52]. The WASI-II is a well-established test that has been validated for use with children, adolescents and adults. The WIAT-III is a standardized comprehensive academic achievement battery [53]. It has strong psychometric properties. The Numerical Operations subtest measured math achievement and the Word Reading subtest measured reading achievement. Standard scores are available for WASI and WIAT scores.

#### Impairment rating scale

The Impairment Rating Scale (IRS) was administered to parents and teachers (IRS) [54]. Parents and teachers indicated the adolescent’s impairment severity in seven domains on a Likert scale ranging from “0 = no problem” to “6 = extreme problem.” The IRS demonstrates strong psychometrics and accurately identifies ADHD-related impairment across settings and informants [54].

#### Diagnostic interview schedule for children (DISC)

The DISC is a structured interview that was administered to assess ADHD, ODD, and CD diagnoses. The DISC queries the presence of each symptom (0 = No, 1 = Yes). The ADHD module contains supplemental probes for symptom-specific impairment [32]. Symptom presence is evaluated for each symptom of ADHD, ODD, and CD using parent reports.

#### Parent academic management scale

The PAMS is a 16-item checklist that measures the frequency of adaptive and maladaptive parental involvement strategies related to adolescent OTP skills [55]. Parents indicate the number of days during the typical school week (0 to 5) that they performed each activity. PAMS possesses strong psychometric properties as evidenced by good internal consistency, concurrent validity, and predictive validity [55]. In 2016, an item was added to the PAMS querying the number of hours the parent spends each week in activities related to the adolescent’s academics.

#### Caregiver strain questionnaire

Parent strain stemming from the parent-adolescent relationship was measured by the 21 item *Caregiver Strain Questionnaire* (CSQ) [56]. The parent indicates how his/her child’s problems affected the parents and family over the past 4 weeks. Responses were scored on a 5-point scale ranging from not at all to very much a problem. The CSQ shows strong psychometric properties and the measure correlates well with other measures of family functioning.

#### Adult ADHD self-report scale

The Adult ADHD Self-Report Scale (ASRS) measured parental ADHD [57]. Eighteen adult-specific ADHD symptoms were rated on a five-point scale (0 = Never to 4 = Very Often). The ASRS correlates highly with clinician ADHD ratings and displays strong internal consistency [57]. Parental ADHD severity is calculated as the mean score of ASRS items.

#### Symptom Checklist-90-revised

The Symptom Checklist-90-Revised (SCL-90-R) is a 90-item broadband scale of adult psychopathology that measures nine symptom domains using a 5-point Likert scale [34]. The SCL-90-R has good internal consistency for each subscale and possesses convergent, discriminant, and predictive validity [58]. Individual items and T-scores from the SCL-90-R are included in the dataset.

#### Patient health Questionnaire-9

The Patient Health Questionnaire-9 depression scale has excellent internal reliability as well as criterion and construct validity [33]. Parents reported on whether they experienced a range of depressive symptoms during the past 2 weeks, rating symptom frequency from 0-not at all to 3-nearly every day.

#### World Health Organization quality of life questionnaire

Parents completed the World Health Organization (WHO) Quality of Life Questionnaire, a multidimensional profile of quality of life for cross cultural use. The English version is self-administered and covers 25 facets of quality of life within six broad domains. It captures positive and negative aspects of quality of life and possesses strong psychometric properties [35].

#### Harmonization

Harmonization of measures followed methods of Bauer (2017) [36] and Curran et al. (2008) [37].

#### Parent depression

Parent depression was assessed by the SCL-90 (studies A and B), PHQ-9 (study C), and the WHO QOL (study D). Specific items from the SCL-90 (13 of 90 items) and the WHO QOL (5 of 26 items) that reflected symptoms of major depression were selected; the PHQ-9 measures the nine DSM symptoms of major depression and all items were included. Each participant’s item scores were combined and recoded to reflect endorsement of the nine DSM-5 major depression symptoms. Nonlinear moderated factor analysis (NLMFA) was used to determine item-specific characteristics (i.e., discrimination and difficulty) within the IRT framework, how those item characteristics vary across study, and to provide each participant with a common-scale score of depression [36]. This harmonized, common-scale score can be used in any analyses involving the total IDA sample.

#### ADHD severity

Adolescent ADHD symptoms were measured using DSM-IV criteria in studies A and B and using DSM-5 criteria in studies C and D; both parent and teacher report of adolescent ADHD symptoms were collected. The DSM-IV-TR criteria (e.g., often fails to give close attention to details or makes careless mistakes in schoolwork, work, or during other activities) omits corresponding adolescent/adult specifiers added to the DSM-5 ADHD criteria (e.g. … overlooks or misses details, work is inaccurate). For each participant, item scores were recoded to reflect endorsement the 18 ADHD symptoms. Since ADHD symptoms are grouped into IN and HI, a two-factor model was used. Nonlinear moderated factor analysis (NLMFA) was used to determine item-specific characteristics (i.e., discrimination and difficulty) within the IRT framework, how those item characteristics vary across study, and to provide each participant with common-scale scores of IN and HI ADHD symptoms [36]. These harmonized, common-scale scores can be used in any analyses involving the total IDA sample. Separate models and scores were created for parent report and teacher report of symptoms.

## Utility and discussion

Our research team has several analyses planned using the ADHD TIDAL dataset. However, numerous opportunities for data analysis exist beyond our specific aims. We believe that pursuing personalized medicine questions for adolescents with ADHD will provide useful information that promotes improved treatment engagement and response—leading to meaningful changes in long-term outcomes. We invite additional research teams to utilize the ADHD TIDAL dataset, which is publicly available for use at the National Institute of Mental Health, National Data Archive (https://nda.nih.gov).

### Investigator aims

Our first aim is to identify clinical and family-risk profiles that divide the heterogeneous population into clinically meaningful subgroups. In doing so, we will identify unobserved groups of individuals who differ from one another on a combination of baseline measures. Latent profile analysis (LPA) will be used to identify treatment-relevant phenotypes and environmental factors based on relevant individual (e.g., gender, race/ethnicity, age, ODD/CD severity, ADHD subtype, depression severity, anxiety severity, organization skills, % of school work turned in, average test/assignment grades, school attendance, school disciplinary incidents, IQ, achievement) and family context variables (e.g., parent education level/SES, parent English skills, parent marital status, parent-teen conflict, parental ADHD, parental well-being, family size). Variables that demonstrate superior psychometric properties when modeled as an observed variable will not be modeled in the context of the latent profiles.

In a second aim, we will examine whether baseline latent profile and observed variables predict treatment engagement and response. The first analyses will describe who is most at risk for treatment disengagement (i.e., medication and psychosocial). Finally, we will examine whether baseline latent profile and observed variables predict treatment response, with primary outcomes (ADHD symptoms, parent-teen conflict, and GPA) as the distal outcomes.

Our third aim will examine heterogeneity in treatment response over time. This aim will identify latent, unobserved groups of individuals who differ from one another in terms of their outcome (ADHD symptoms, parent-teen conflict, GPA, OTP problems) trajectory over time. This analysis will allow us to examine this heterogeneity and determine if clinical profile, family context, adjunctive supports (e.g., medication status, parent involvement, class placement, school accommodations), and treatment characteristics (e.g., time of year, setting of treatment, % of treatment attended, content of treatment) predict treatment response.

Our fourth aim will be to identify key treatment mediators and moderators of the relationship between treatment group and change in key outcomes (ADHD symptoms, GPA, parent-teen conflict, OTP problems). Potential mediators of the treatment effect on outcomes include teen organization skills, parent contingency management, parent-teen conflict, and parental well-being. Potential moderators of the treatment effect on outcomes include individual, treatment, and family variables (as noted above).

### Sensitivity analysis

Given the fixed sample size, we present sensitivity analyses that provide the smallest effect that can be detected, rather than power analyses. For latent profile analyses and growth mixture models proposed in aims 1 through 3, simulation studies indicate that the Bayesian Information Criterion and bootstrap likelihood-ratio test perform best at determining the correct number of classes [59, 60]. These studies show that these measures have at least 80% power to detect the correct number of classes when sample size is greater than 500, if at least 8 indicators of the latent class are used. With a combined sample size of 854, we expect to have sufficient power to correctly identify emergent latent classes based on baseline variables. In aim 4 we will examine questions of moderated mediation with a sample size of 854. For moderation analyses, treatment level variables, the individual level moderator, and their interaction will predict change over time in the outcome variable. A simplified power analysis for a repeated-measures ANOVA design with an interaction between group and time (a much less powerful model than a latent growth model) suggests that for *N* = 854, 9 treatment groups (across all four studies), and 3 measurement occasions, the required effect size is approximately d = 0.12, a very small effect. In previous analyses of individual studies included in this IDA, we found effect sizes of *d* = 0.5 or higher for moderation of the treatment effect on ADHD symptoms (i.e., Sibley et al., 2016). For the mediation analyses, we used the tables generated by Fritz and MacKinnon [61]. With a sample size of *N* = 854 and using the preferred method of bootstrap confidence intervals, we have greater than 80% power to detect even small effects for both the treatment to mediator and mediator to outcome slope. In previous analyses of individual studies included in this IDA, we found effect sizes of *d* = 0.5 or higher for the moderating effect of treatment on ADHD symptoms (equivalent to the effect of a mediating variable on the outcome slope).

### Additional research directions

The ADHD TIDAL dataset is suitable for examining treatment outcome questions that expand on those noted above by selecting independent and dependent variables, moderators, and mediators that our team did not incorporate into our planned analyses. Given the broad age range of participants, cross-sequential analyses with the ADHD TIDAL dataset could reveal important information about the nature of ADHD symptoms and related impairments. The samples demographic diversity also may support research questions related to gender, cultural, or socioeconomic differences in the expression of ADHD. The broad range of data available in the ADHD TIDAL dataset also may help research teams estimate effect sizes for power analyses and conduct pilot analyses prior to data collection studies, as well as further integration with existing datasets.

## Data Availability

The datasets generated during and/or analysed during the current study are available in the National Institute of Mental Health, National Data Archive repository, https://nda.nih.gov.

## Abbreviations

ADHD: Attention deficit/hyperactivity disorder
IDA: Integrative data analysis;
GPA: Grade point average
TIDAL: Teen integrative data analysis longitudinal
DISC: Diagnostic interview schedule for children
STAND: Supporting teens’ autonomy daily
STP-A: Summer treatment program-adolescent
STAND-G: Supporting teen’s autonomy daily-group
UC: Usual care
AAPC: Adolescent academic problems checklist
OTP: Organization, time management, and planning
CBQ: Conflict behavior questionnaire
DBD-RS: Disruptive behavior disorder rating scale
IN: Inattention
HI: Hyperactivity/impulsivity
DSM: Diagnostic and statistical manual
CBCL: Child behavior checklist
YSR: Youth self report
WASI: Wechsler abbreviated scale of intelligence
IQ: Intelligence quotient
WIAT: Wechsler Individual achievement test
IRS: Impairment rating scale
PAMS: Parent academic management scale
CSQ: Caregiver strain questionnaire
ASRS: Adult ADHD self report scale
SCL-90-R: Symptoms checklist-90-revised
PHQ-9: Patient health questionnaire-9
WHO: World health organization
QOL: Quality of life
NLMFA: Nonlinear moderated factor analysis
ODD: Oppositional defiant disorder
CD: Conduct disorder
